# Supplementary material for: Dynamics and clinical relevance of maternal mRNA clearance during the oocyte-to-embryo transition in humans
Source: Nat Commun. 2020 Oct 1;11:4917. doi: 10.1038/s41467-020-18680-6 (PMC7530992; doi:10.1038/s41467-020-18680-6)
Supplement: Supplementary file 1 — Supplementary Information [file 41467_2020_18680_MOESM1_ESM.pdf]

Dynamics and Clinical Relevance of Maternal mRNA Clearance during the Oocyte-to-embryo Transition in Humans

Sha QQ et al.

Supplementary Materials:

Supplementary Figure 1-3
Supplementary Table 1-7

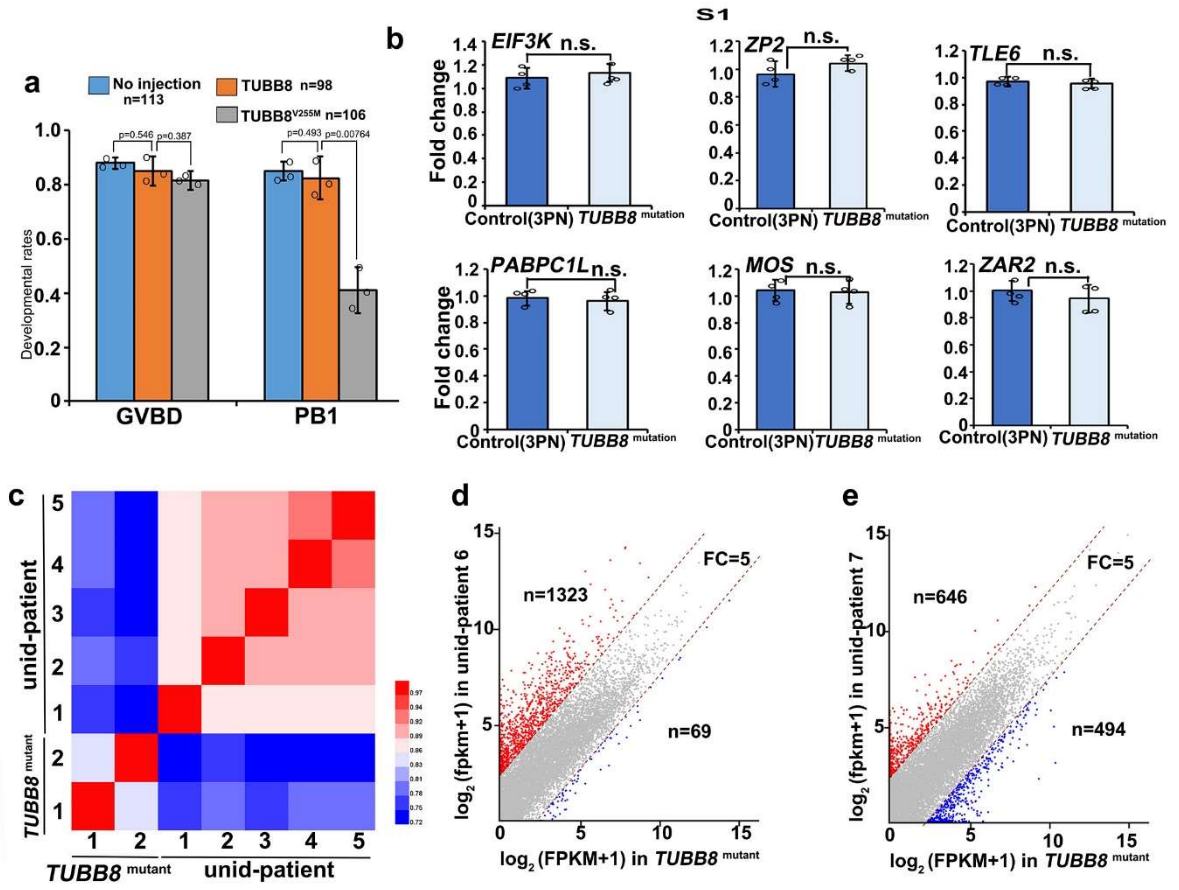

Supplementary Figure 1: Characterization of M-decay during the maternal-to-zygotic transition in humans. a: Rates of germinal vesicle breakdown (GVBD) and polar body 1 (PB1) emission in oocytes that were cultured *in vitro*. Fully grown GV oocytes were collected from PMSG-primed (44 h) mice and were microinjected with mRNAs that encode either wild type or mutant (V255M) TUBB8. Error bars, SEM. P value by a two-tailed Student's *t*-test. n.s.: non-significant. The numbers of analyzed oocytes are indicated (n). **b:** One way ANOVA test comparing differences of M-decay transcripts between the 3PN zygotes at day 1 after IVF and arrested zygotes of TUBB8-mutated patients at day 3 after IVF. Data are presented as mean values +/- SEM. n.s.: non-significant. n = 3 independent experiments. **c:** A heatmap of the Spearman correlation coefficients of total transcripts among arrested zygotes that were derived from *TUBB8*-mutated patients and unid-patients. **d-e:** A scatter plot is shown, which compares transcripts of arrested zygotes 3 days after IVF, as derived from *TUBB8*-mutated and unid-patients. Transcripts decreased or increased more than 5 folds in samples of unid-patients compared to in *TUBB8*-mutated samples, which were highlighted in blue or red, respectively. n, gene number; FC, fold change.

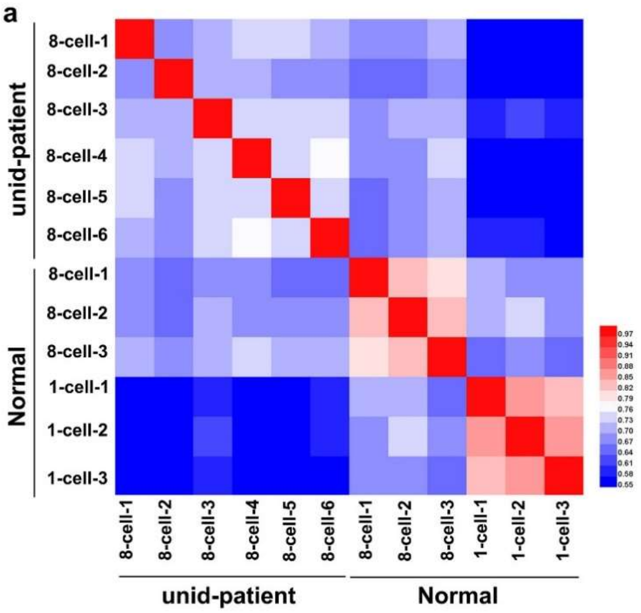

b

Fold change (FC)	Down-regulated	Up-regulated
1<FC<=2	9321	16064
2<FC<=5	2442	4322
5<FC<=10	405	699
FC>10	121	269

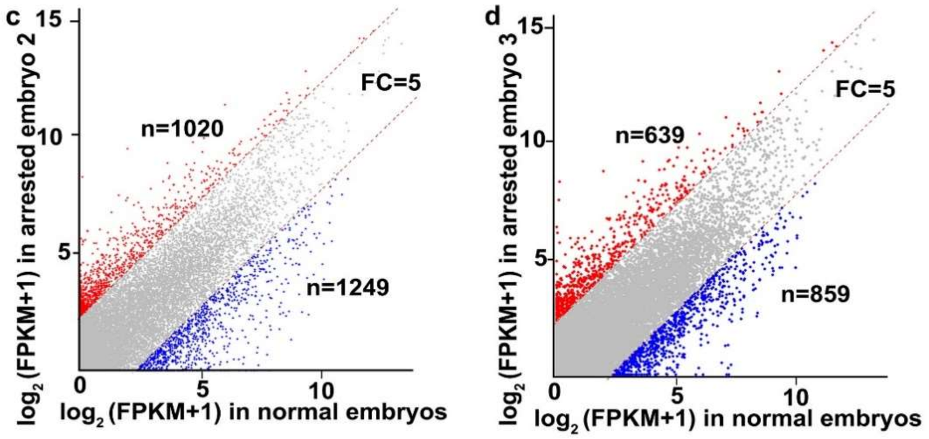

Supplementary Figure 2: Characterization of Z-decay during the maternal-to-zygotic transition in humans. a: A heatmap of the Spearman correlation coefficients of total transcripts among the normal and 8-cell stage-arrested embryos. **b:** Number of transcripts that are upregulated and downregulated in arrested embryos compared to in normal embryos at the 8-cell stage. **c-d:** Scatter plot is shown, which compares transcripts in the 8-cell stage-arrested embryos 5 days after IVF, as derived from unid-patients (#2 and #3) with normal 8-cell embryos. Transcripts decreased or increased more than 5 folds in samples of unid-patients compared to normal samples, which were highlighted in blue or red, respectively. n, gene number; FC, fold change.

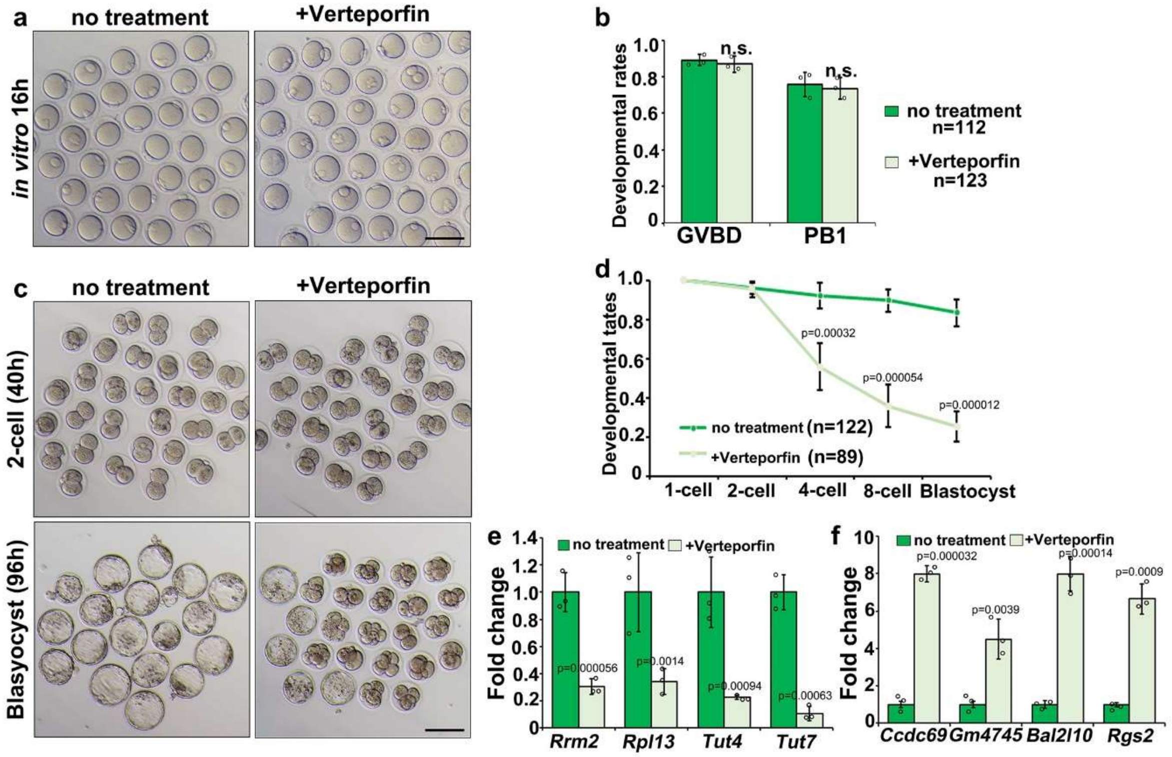

Supplementary Figure 3: Effect of YAP inhibitor verteporfin on mouse early embryo development. a-b: Representative images (**a**) and rates of germinal vesicle breakdown (GVBD) and PB1 emission (**b**) of *in vitro* matured mouse oocytes. Fully grown GV oocytes were cultured with or without the presence of 1 μM verteporfin for 18 h. Scale bar = 100 μm. All images looked like this in 3 independently repeated experiments. The numbers of analyzed oocytes are indicated (n). **c-d:** Representative images (**c**) and rates of preimplantation embryo development (**d**) of *in vitro* cultured mouse embryos. Zygotes were cultured with or without the presence of verteporfin for 96 h. Scale bar = 100 μm. **e-f:** RT-qPCR results showing levels of indicated transcripts in the 2-cell embryos with or without verteporfin treatment. n = 3 independent experiments.

In **b**, **d**, **e**, and **f**, Data are presented as mean values +/- SEM. P by a two-tailed Student's *t*-test.

Supplementary Tables

Supplementary Table 1: Spearman correlation coefficients among arrested zygotes from *TUBB8*-mutated patients and unid-patients 3 days after IVF.

		TUBB8 mutant		**unid-patient**				
		**1**	**2**	**1**	**2**	**3**	**4**	**5**
**unid-patient**	**5**	0.789547	0.737685	0.875441	0.91486	0.909213	0.924252	1
	**4**	0.787375	0.735872	0.877783	0.915648	0.909887	1	0.924252
	**3**	0.775127	0.723919	0.879238	0.91942	1	0.909887	0.909213
	**2**	0.800907	0.751266	0.88005	1	0.91942	0.915648	0.91486
	**1**	0.768366	0.722768	1	0.88005	0.879238	0.877783	0.875441
TUBB8 mutant	**2**	0.843335	1	0.722768	0.751266	0.723919	0.735872	0.737685
	**1**	1	0.843335	0.768366	0.800907	0.775127	0.787375	0.789547

Supplementary Table 2: One way ANOVA analyzes differences of M-decay transcripts between the 3PN zygotes at day 1 after IVF and arrested zygotes of *TUBB8*-mutated patients at day 3 after IVF.

Gene name	*TUBB8* *mutation* 1-cell (No.)	Unid-patient 1-cell (No.)	Mean Difference	Std. Error	Significance. (*p-value*)	95% Confidence Interval	
						Lower Bound	Upper Bound
EIF3K	1-4	1-9	-8.626734043*	3.304510959	.01893	-15.63198434	-1.62148375
		8-15	-1.800197889	3.382274797	.60187	-8.97030016	5.36990438
MOS	1-4	1-9	-5.556771669*	2.371942225	.03240	-10.58506456	-.52847878
		8-15	-.719590681	2.427760267	.77074	-5.86621254	4.42703117
PABPC1L	1-4	1-9	-.710011476	.870588762	.42673	-2.55557721	1.13555425
		8-15	-.737950435	.891076006	.41976	-2.62694718	1.15104631
TLE6	1-4	1-9	-2.367973948*	1.037751012	.03653	-4.56790782	-.16804008
		8-15	-1.469448530	1.062172024	.18553	-3.72115263	.78225557
ZAR1L	1-4	1-9	-9.465412279*	2.545496618	.00187	-14.86162405	-4.06920051
		8-15	-.177453900	2.605398851	.94654	-5.70065273	5.34574493
ZP2	1-4	1-9	-10.791933733*	3.937407254	.01450	-19.13886424	-2.44500323
		8-15	-1.313376593	4.030064808	.74873	-9.85673233	7.22997915
CNOT6L	1-4	1-9	.571257799*	.132058448	.00052	.29130639	.85120920
		8-15	.347036083*	.135166131	.02066	.06049669	.63357548
CNOT7	1-4	1-9	.381401890	.251510890	.04891	-.15177738	.91458116
		8-15	.484749939	.257429603	.07801	-.06097644	1.03047632
BTG4	1-4	1-9	.868608342*	.173143689	.00013	.50156012	1.23565656
		8-15	.392836803*	.177218215	.04148	.01715097	.76852264

*: The mean difference is significant at the 0.05 level.

Supplementary Table 3: Spearman correlation coefficients among normal embryos and embryos from unid-patients.

		unid-patient						normal					
		8-cell-1	8-cell-2	8-cell-3	8-cell-4	8-cell-5	8-cell-6	8-cell-1	8-cell-2	8-cell-3	1-cell-1	1-cell-2	1-cell-3
unid-patiernt	8-cell-1	1	0.6927	0.7256	0.7413	0.741	0.7249	0.6756	0.686	0.7244	0.5703	0.5769	0.5501
	8-cell-2	0.6927	1	0.7226	0.7141	0.6973	0.6985	0.646	0.654	0.6786	0.5613	0.5697	0.5517
	8-cell-3	0.7256	0.7226	1	0.7471	0.7302	0.7593	0.6983	0.7038	0.7275	0.6048	0.6111	0.5886
	8-cell-4	0.7413	0.7141	0.7471	1	0.747	0.7814	0.6783	0.6949	0.7318	0.5685	0.5775	0.5494
	8-cell-5	0.741	0.6973	0.7302	0.747	1	0.7496	0.6687	0.6789	0.7174	0.5648	0.576	0.5471
	8-cell-6	0.7249	0.6985	0.7593	0.7814	0.7496	1	0.669	0.6934	0.7215	0.5854	0.5962	0.565
normal	8-cell-1	0.6756	0.646	0.6983	0.6783	0.6687	0.669	1	0.832	0.806	0.7088	0.6967	0.6722
	8-cell-2	0.686	0.654	0.7038	0.6949	0.6789	0.6934	0.832	1	0.8441	0.7293	0.7308	0.6985
	8-cell-3	0.7244	0.6786	0.7275	0.7318	0.7174	0.7215	0.806	0.8441	1	0.6676	0.678	0.6449
	1-cell-1	0.5703	0.5613	0.6048	0.5685	0.5648	0.5854	0.7088	0.7293	0.6676	1	0.862	0.845
	1-cell-2	0.5769	0.5697	0.6111	0.5775	0.576	0.5962	0.6967	0.7308	0.678	0.862	1	0.8661
	1-cell-3	0.5501	0.5517	0.5886	0.5494	0.5471	0.565	0.6722	0.6985	0.6449	0.845	0.8661	1

Supplementary Table 4: One way ANOVA test comparing differences at the 8-cell stage between normal and Unid-patients.

Gene name	normal 8-cell (No.)	Unid-patient 8-cell (No.)	Mean Difference	Std. Error	Significance. (*p-value*)	95% Confidence Interval	
						Lower Bound	Upper Bound
MYC	1-3	1-8	.554055213*	.109104118	.00017	.32005015	.78806027
		9-14	.354318975*	.113955535	.00769	.10990866	.59872929
TEAD4	1-3	1-8	.525185599*	.186574757	.01377	.12502254	.92534865
		9-14	.490985642*	.194870978	.02452	.07302896	.90894232
TUT7	1-3	1-8	.639879139*	.132556699	.00027	.35557329	.92418498
		9-14	.547490746*	.138450957	.00144	.25054298	.84443852
TUT4	1-3	1-8	.200576445	.262568649	.45761	-.36257730	.76373019
		9-14	.170790507	.274244010	.54345	-.41740439	.75898541
CNOT7	1-3	1-8	-1.183387142	.575320096	.04883	-2.41732603	.05055174
		9-14	-.533498605	.600902242	.38963	-1.82230574	.75530852
SKP1	1-3	1-8	-5.78704473*	1.311881242	.00059	-8.60075016	-2.97333931
		9-14	-.932695502	1.370215269	.50717	-3.87151497	2.00612397
SUV39H2	1-3	1-8	-4.70874980*	1.091520943	.00071	-7.04982939	-2.36767022
		9-14	-1.107299476	1.140056443	.34790	-3.55247736	1.33787841
CENPJ	1-3	1-8	-3.73175434*	1.041275673	.00299	-5.96506854	-1.49844014
		9-14	-.846452662	1.087576970	.44935	-3.17907327	1.48616795

*. The mean difference is significant at the 0.05 level.

Supplementary Table 5: Quality control of RNA-seq results among arrested zygotes from *TUBB8*-mutated patients and unid-patients 3 days after IVF.

Sample	Total reads	Mapping efficiency	Uniquely mapping efficiency
unid-patient-1	29521076	96.15	99.18
unid-patient-2	31915446	96	99.17
unid-patient-3	31744544	96.4	98.89
unid-patient-4	31410860	96.54	99.06
unid-patient-5	27700306	95.91	98.94
TUBB8-mutant-1	26214748	98.35	95.63
TUBB8-mutant-2	29871274	99.12	94.8

Supplementary Table 6: Quality control of RNA-seq results among arrested 8-cell embryos from unid-patients.

Sample	Total reads	Mapping efficiency	Uniquely mapping efficiency
Patient 8-cell-1	29759666	98.84	93.59
Patient 8-cell-2	29202842	99.08	94.22
Patient 8-cell-3	27922930	99.31	94.77
Patient 8-cell-4	28250132	98.68	93.44
Patient 8-cell-5	28067700	98.66	93.14
Patient 8-cell-6	28602640	98.67	93.53

Supplementary Table 7: Primer sequences.

Primer name	Genes targeted	Application	Sequences (5'-3')
Eif3k -F	*Eif3k*	qPCR (mouse)	5'-CGACAGGTACAATCCTGAGAAC-3'
Eif3k -R			5'-GCCTCCAGATCATAGGCATTC-3'
Pabpc1l -F	*Pabpc1l*	qPCR (mouse)	5'-GCTTCTCGTGCCACATAGAGGAAC-3'
Pabpc1l -R			5'-AGCTCCAGGTTGTCCAGCTCC-3'
Kpna2-F	*Kpna2*	qPCR (mouse)	5'-ATGTCCACGAACGAGAATGCT-3'
Kpna2-R			5'-AAGGAGCTGACGTTTCTTCTTTT-3'
Zp2-F	*Zp2*	qPCR (mouse)	5'-GCAGCTGGAGCTCTTGTTCT-3'
Zp2-R			5'-TCCATTGTCCAAAGTCCACA-3'
Mos -F	*Mos*	qPCR (mouse)	5'- CGCCACGACAACATAGTTCG -3'
Mos -R			5'- ACCGTAGATGACTTGGTGTAGAG -3'
Rps2-F	*Rps2*	qPCR (mouse)	5'- GCCACCTTTGATGCCATCT -3'
Rps2-R			5'- TGTGGTAGCCACAGCTGGA -3'
Gapdh-R	*Gapdh*	qPCR (mouse)	5'-ACACTGAGGACCAGGTTGTCTC-3'
Gapdh -R			5'-TACTCCTTGGAGGCCATGTAG-3'
PABPC1L-F	*PABPC1L*	qPCR (human)	5'-CCTCCAAAGCAAAATCCAGTG-3'

PABPC1L-R			5’-GAAGACAGAAACAGACAACCA-3’
MOS -F	*MOS*	qPCR (human)	5’- TCACTAGATGTTGTGAACGGC -3’
MOS -R			5’- TCAGAGCAACCGAAGTCACTAA-3’
ZP2-F	*ZP2*	qPCR (human)	5’- CTTGCCACGGGTCTTCTCTG -3’
ZP2-R			5’- CACCAACCTCAATGCTCCATC -3’
ZAR2-F	*ZAR2*	qPCR (human)	5’- TGCATTTCTGGAACGAACAAGG-3’
ZAR2-R			5’- TGACATTGGATTGCTTCTACTCG-3’
TLE6-F	*TLE6*	qPCR (human)	5’-CCTGTGCAGGTCTCAACTG-3’
TLE6-R			5’- TGACTCCATCAGGATAACCCTTG-3’
SKP1-F	*SKP1*	qPCR (human)	5’- GACCATGTTGGAAGATTTGGGA-3’
SKP1-R			5’- TGCACCACTGAATGACCTTTT-3’
CENPJ -F	*CENPJ*	qPCR (human)	5’- ACCCGTCCAACATTTTATCCC-3’
CENPJ -R			5’- ACGCACGTTTTGAAGTTAAGACA-3’
SUV39H2-F	*SUV39H2*	qPCR (human)	5’- TACTCGTCTTCCCCGAATAGC-3’
SUV39H2-R			5’-GGCTGTGGTCAATAGAATCTGAA-3’
BTG4-F	*BTG4*	qPCR (human)	5’-TTATGCCGTTAGTAGAGCCTCA-3’
BTG4-R			5’-GCCTGTAACACTTAGGATGCTTC-3’
CNOT7-F	*CNOT7*	qPCR (human)	5’-TGGTTGTCATTTCATAGCGGTT-3’
CNOT7-R			5’-CCACCTCCTGTAATCCACCT-3’
CNOT6L-F	*CNOT6L*	qPCR (human)	5’-AGATGGAACCCGAAAGCTACT-3’
CNOT6L-R			5’-TAACCGTGAATGATGCTGACG-3’
PAN2-F	*PAN2*	qPCR (human)	5’-CAGCAGCACTCTACTCGTTGG-3’
PAN2-R			5’-GTGTGGCCGCAGAAGAAGAA-3’
TUT4-F	*TUT4*	qPCR (human)	5’-TGAGTGCAGGAAACGATATGGC-3’
TUT4-R			5’-GAAAGGCTAACACCAAGGGAATA-3’
TUT7-F	*TUT7*	qPCR (human)	5’-ATGGCTGTCTGATGAACATACTG-3’
TUT7-R			5’-TGTCTTGCCACCTATAACCATCT-3’
TEAD4-F	*TEAD4*	qPCR (human)	5’-GGACACTACTCTTACCGCATCC-3’
TEAD4 -R			5’-TCAAAGACATAGGCAATGCACA-3’
DUSP7-F	*DUSP7*	qPCR (human)	5’-GACGTGCTCGGCAAGTATG-3’
DUSP7-R			5’-GGATCTGCTTGTAGGTGAACTC-3’
