# Supplementary material for: Dynamics and clinical relevance of maternal mRNA clearance during the oocyte-to-embryo transition in humans
Source: Nat Commun. 2020 Oct 1;11:4917. doi: 10.1038/s41467-020-18680-6 (PMC7530992; doi:10.1038/s41467-020-18680-6)
Supplement: Supplementary file 2 — Reporting Summary [file 41467_2020_18680_MOESM2_ESM.pdf]

nature research

Corresponding author(s): Heng-Yu Fan

Last updated by author(s): Aug 7, 2020

Reporting Summary

Nature Research wishes to improve the reproducibility of the work that we publish. This form provides structure for consistency and transparency in reporting. For further information on Nature Research policies, see our Editorial Policies and the Editorial Policy Checklist.

Statistics

For all statistical analyses, confirm that the following items are present in the figure legend, table legend, main text, or Methods section.

n/a	Confirmed	
☐	☒	The exact sample size (n) for each experimental group/condition, given as a discrete number and unit of measurement
☐	☒	A statement on whether measurements were taken from distinct samples or whether the same sample was measured repeatedly
☐	☒	The statistical test(s) used AND whether they are one- or two-sided<br>*Only common tests should be described solely by name; describe more complex techniques in the Methods section.*
☐	☒	A description of all covariates tested
☐	☒	A description of any assumptions or corrections, such as tests of normality and adjustment for multiple comparisons
☐	☒	A full description of the statistical parameters including central tendency (e.g. means) or other basic estimates (e.g. regression coefficient) AND variation (e.g. standard deviation) or associated estimates of uncertainty (e.g. confidence intervals)
☐	☒	For null hypothesis testing, the test statistic (e.g. F, t, r) with confidence intervals, effect sizes, degrees of freedom and P value noted<br>*Give P values as exact values whenever suitable.*
☒	☐	For Bayesian analysis, information on the choice of priors and Markov chain Monte Carlo settings
☒	☐	For hierarchical and complex designs, identification of the appropriate level for tests and full reporting of outcomes
☒	☐	Estimates of effect sizes (e.g. Cohen's d, Pearson's r), indicating how they were calculated

Our web collection on statistics for biologists contains articles on many of the points above.

Software and code

Policy information about availability of computer code

Data collection	Trimmomatic (v0.35)
Data analysis	STAR aligner (v2.5.2b); HTSeq (v0.6.1p1)

For manuscripts utilizing custom algorithms or software that are central to the research but not yet described in published literature, software must be made available to editors and reviewers. We strongly encourage code deposition in a community repository (e.g. GitHub). See the Nature Research guidelines for submitting code & software for further information.

Data

Policy information about availability of data

All manuscripts must include a data availability statement. This statement should provide the following information, where applicable:

- Accession codes, unique identifiers, or web links for publicly available datasets
- A list of figures that have associated raw data
- A description of any restrictions on data availability

RNA-seq data have been deposited in the NCBI Gene Expression Omnibus database under accession code PRJNA603589.

Field-specific reporting

Life sciences study design

All studies must disclose on these points even when the disclosure is negative.

Sample size	No sample size calculation was performed. Sample sizes were chosen based on oocytes or zygotes available from superovulation of mouse or human patients. These sample sizes are sufficient because it reflects the normal sizes of oocytes and zygotes in natural reproduction process.
Data exclusions	No data were excluded from the analyses.
Replication	All experiments were independently repeated for more than 3 times with the consistent results.
Randomization	All allocations were random in this study.
Blinding	The investigators were blinded to group allocation during data collection and analysis.

Reporting for specific materials, systems and methods

We require information from authors about some types of materials, experimental systems and methods used in many studies. Here, indicate whether each material, system or method listed is relevant to your study. If you are not sure if a list item applies to your research, read the appropriate section before selecting a response.

Materials & experimental systems

n/a	Involved in the study	
☐	☒	Antibodies
☒	☐	Eukaryotic cell lines
☒	☐	Palaeontology and archaeology
☐	☒	Animals and other organisms
☒	☐	Human research participants
☒	☐	Clinical data
☒	☐	Dual use research of concern

Methods

n/a	Involved in the study	
☒	☐	ChIP-seq
☒	☐	Flow cytometry
☒	☐	MRI-based neuroimaging

Antibodies

Antibodies used	BTG4 Abcam (ab206914); CNOT7 Abcam (ab195587); FITC-α-tubulin Sigma (F2168); YAP1 Cell Signaling (#14074)
Validation	Recombinant rabbit monoclonal anti-BTG4 (Specificity: mouse, human);<br>Recombinant rabbit monoclonal anti-CNOT7 (Specificity: mouse, human);<br>Mouse monoclonal FITC-α-tubulin (Specificity: almost all vertebrate);<br>Recombinant rabbit monoclonal anti-YAP (Specificity: mouse, rat, human);

Animals and other organisms

Policy information about studies involving animals; ARRIVE guidelines recommended for reporting animal research

Laboratory animals	mouse, C57BL6 strain, female 3-4 weeks old.
Wild animals	No wild animals were used in the study.
Field-collected samples	No field collected samples were used in the study.
Ethics oversight	The experimental protocols that involved mice were approved by the Zhejiang University Institutional Animal Care and Research Committee (Approval # ZJU20170014), and mouse care and use was performed in accordance with the relevant guidelines and regulations.

Note that full information on the approval of the study protocol must also be provided in the manuscript.
